# Short-Lasting Unilateral Neuralgiform Headache Attacks With Conjunctival Injection and Tearing (SUNCT) Across Two Decades: Longitudinal Characterization and Multimodal Management of a Refractory Case

**DOI:** 10.7759/cureus.112845

**Published:** 2026-07-17

**Authors:** Gabriela P Silva, Marina R Nannini, Carlos A Bordini, Hilton M Junior

**Affiliations:** 1 Neurology, Municipal University Centre of Franca, Franca, BRA; 2 Neurology, Pontifical Catholic University of Campinas, Campinas, BRA

**Keywords:** case report, chronic pain, sunct, trigeminal autonomic cephalalgia, trigeminal neuralgia

## Abstract

Short-lasting Unilateral Neuralgiform headache attacks with Conjunctival injection and Tearing (SUNCT) is a rare and often refractory primary headache disorder marked by extreme therapeutic complexity. We present the case of a 21-year follow-up of a 45-year-old woman with a 31-year clinical history of severe periorbital pain, which began in adolescence and was associated with a neurovascular conflict involving the nervus intermedius. She was first evaluated at age 24 after being referred by a neurosurgeon due to severe, right-sided, unilateral periorbital pain, mainly affecting the V1 territory. Diagnostic workup showed a neurovascular conflict involving the nervus intermedius. Throughout evolution, autonomic symptoms such as tear production, conjunctival redness, and eyelid swelling have been observed. The clinical course demonstrated prominent autonomic symptoms and resistance to multiple pharmacological and surgical interventions, with partial relief achieved only through a multimodal regimen of gabapentin, clonazepam, and indomethacin. This case illustrates the chronic nature of the pathology and emphasizes the importance of long-term longitudinal follow-up and multidisciplinary management in treatment-resistant presentations.

## Introduction

Short-lasting Unilateral Neuralgiform headache attacks with Conjunctival injection and Tearing (SUNCT) is a rare primary headache disorder classified as a trigeminal autonomic cephalalgia according to the International Classification of Headache Disorders, third edition [1]. It is characterized by brief attacks of strictly unilateral head pain lasting from 1 to 600 seconds, typically accompanied by prominent ipsilateral cranial autonomic symptoms such as conjunctival injection and lacrimation [1,2]. Attacks may occur multiple times per day and can be highly disabling due to their frequency and intensity. Epidemiological studies indicate that SUNCT is an uncommon condition, with population-based data demonstrating a very low prevalence compared with other primary headache disorders [3].

The pathophysiology of SUNCT is not fully understood but is believed to involve activation of the trigeminal-autonomic reflex, leading to simultaneous trigeminal nociceptive activation and cranial parasympathetic outflow. Functional and clinical studies suggest that hypothalamic activation contributes to the generation of attacks, a mechanism shared with other trigeminal autonomic cephalalgias [4,5]. The clinical spectrum also overlaps with related conditions such as Short-lasting Unilateral Neuralgiform headache attacks with cranial Autonomic symptoms (SUNA) and other short-lasting neuralgiform headaches, which can create diagnostic challenges in clinical practice [2].

In addition to primary forms, symptomatic trigeminal autonomic cephalalgias have been described in association with structural lesions or other neurological disorders, reinforcing the importance of careful evaluation when establishing the diagnosis [6,7]. Therapeutic management can be challenging, as responses to available pharmacological treatments vary considerably. Although lamotrigine is frequently considered a first-line therapy, many patients require multiple treatment strategies due to refractory or recurrent symptoms [2,8].

Long-term observational data on SUNCT remain scarce due to the condition's rarity. Most available studies report limited follow-up periods, and the disorder's natural history over decades is poorly characterized. In this report, we present the longitudinal clinical course, therapeutic management, and complications observed over 21 years in a patient with refractory SUNCT, representing one of the longest documented follow-ups of this condition to date and contributing to a better understanding of its long-term evolution and management. This report, by documenting more than two decades of follow-up in a patient with refractory SUNCT, emphasizes the challenges of long-term management, the importance of careful individualized therapeutic decision-making, and ongoing observation in patients with this rare primary headache disorder.

## Case presentation

A 45-year-old woman experiences severe right-sided periorbital pain, primarily affecting the ophthalmic division (V1) of the trigeminal nerve. Her symptoms began at age 14, and she first sought our medical attention in 2004 when she was 24 years old.

Initial investigations included computed tomography, brain magnetic resonance imaging, venous-phase MR angiography, and cerebrospinal fluid analysis, all of which were unremarkable. Arterial MR angiography showed a neurovascular conflict between the nervus intermedius and a vascular loop. The patient was refractory to multiple drugs, including amitriptyline 25 mg, propranolol 60 mg, pizotifen 1 mg, divalproex, methadone, carbamazepine, and their combinations. She underwent sinus surgery without benefit and two thermocoagulation procedures of V1 in 1997 and 1999, with partial relief lasting 6-12 months. Afterward, microvascular decompression was performed in 2002, resulting in symptom relief for approximately 1 year. Surgical complications were severe neuralgiform pain along the craniotomy margin and painful anesthesia in the V1 territory.

**Figure 1:**
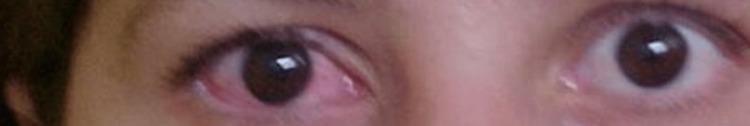
Signs of lacrimation, conjunctival injection, and eyelid edema. Image reproduced with patient's authorization and consent.

In 2004, during the initial evaluation, the patient reported experiencing daily attacks of strictly right-sided periorbital pain. These attacks occurred 8 to 20 times per day, lasted 2 to 8 minutes, and reached a maximum intensity of 10/10. Associated symptoms included lacrimation, conjunctival injection, and eyelid edema, as illustrated in Figure 1. During attacks, the patient was occasionally restless and distressed. Between attacks, she presented continuous nonpulsatile pain (4/10), not only in the periorbital region but also in the right temporal area. Examination revealed marked right conjunctival injection and eyelid edema, intense pain along the surgical scar, and attacks triggered by digital pressure over the right greater occipital nerve. Hypoesthesia in the V1 territory was also noted.

**Figure 2:**
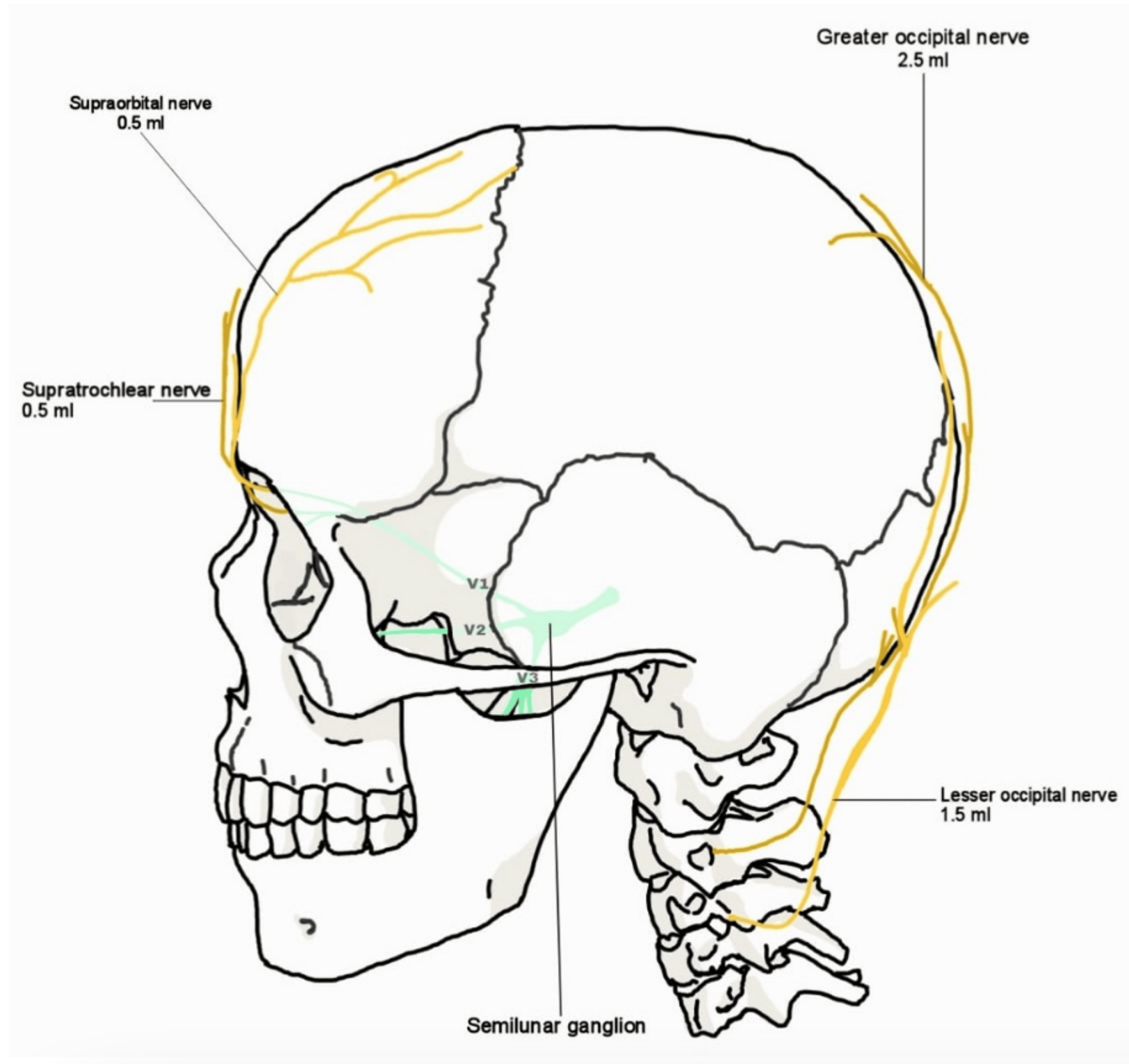
Anatomical schematic depicting the right supraorbital, supratrochlear, greater occipital, and lesser occipital nerves, with the respective volumes of anesthetic/corticosteroid used in the block (0.5 ml, 0.5 ml, 2.5 ml, and 1.5 ml). Created by the authors using Samsung Notes (Samsung Electronics Co., Ltd., Suwon, South Korea). No generative artificial intelligence (AI) tools or AI-assisted features were used to create this figure.

Over the following 2 years, local nerve blocks using lidocaine 2% and dexamethasone 4 mg were administered at painful points, employing the "follow-the-pain" strategy and at the greater occipital nerve, resulting in complete relief for 1 week. The 5 ml syringe was prepared with 4 ml of lidocaine 2% without vasoconstrictor and 1 ml of betamethasone dipropionate 5 mg and betamethasone sodium phosphate 2 mg. The injection was applied to the right supraorbital, supratrochlear, greater occipital, and lesser occipital nerves, respectively, at 0.5 ml, 0.5 ml, 2.5 ml, and 1.5 ml. In addition, 0.5 ml was applied to each painful point along the surgical margin, "following the pain." The anatomical distribution of the blocked nerves and the injected volumes are shown in Figure 2.

Upon recurrence of attacks, myoclonus in the levator labii superioris alaeque nasi muscle was observed, associated with severe dysesthesia in the same territory. Attacks were refractory to subcutaneous sumatriptan. Repeat nerve blocks were performed, and treatment with gabapentin 400 mg TID, indomethacin 50 mg BID, amitriptyline 25 mg BID, and clonazepam 1 mg TID was initiated. Pain and facial myoclonus were controlled. Sporadic attacks, when present, were managed with sublingual clonazepam 0.25 mg. The patient remained stable for approximately 2 years, after which excruciating attacks recurred, and continuous ocular pain with conjunctival hyperemia developed. Usual symptoms were controlled with nerve blocks and an increase in gabapentin to 400 mg QID; however, intense ocular pain persisted, and ophthalmologic evaluation revealed a corneal ulcer, which resolved within 15 days.

Between 2009 and 2013, symptom control was maintained using gabapentin and clonazepam, while onabotulinum toxin injections were administered every 3 to 4 months, at first 75 U following the pain and later at sites specified in the PREEMPT protocol [9]. Occasional recurrences were managed with nerve blocks, as specified above, although highly uncomfortable dysesthesia (painful anesthesia) persisted. A new MR angiography showed no vascular compression. In March 2010, the patient underwent hysterosalpingo-oophorectomy. Four days after surgery, she developed severe pain on the right side of the surgical incision, similar to the previous pain, refractory to morphine and responsive to local anesthetic blockade.

In 2011, typical SUNCT attacks recurred (more than 50 attacks per day, duration 3 to 5 minutes), accompanied by lacrimation, conjunctival injection, and myoclonus. The patient was intolerant of lamotrigine and indomethacin. Multiple nerve blocks, including 0.5 ml at the C4 level, next to the spinous process, resulted in improvement.

Treatment with haloperidol 1 mg TID and lithium carbonate 300 mg BID was initiated, while gabapentin 400 mg TID and clonazepam 1 mg TID were maintained, leading to a good clinical response and remission lasting 3 years. The patient gradually tapered gabapentin on her own and discontinued botulinum toxin therapy.

During long-term follow-up through 2025, the patient experienced an episode of ophthalmic herpes zoster on the right side in 2020 without recurrence of SUNCT attacks, and duloxetine 60 mg/day was initiated. Painful anesthesia persisted but was partially controlled with duloxetine and pregabalin. In 2025, the patient was contacted and reported having gradually discontinued all medications except duloxetine 60 mg/day, beginning in 2021. She has remained free of typical SUNCT attacks for several years, with only mild residual pain and good quality of life.

## Discussion

**Figure 3:**
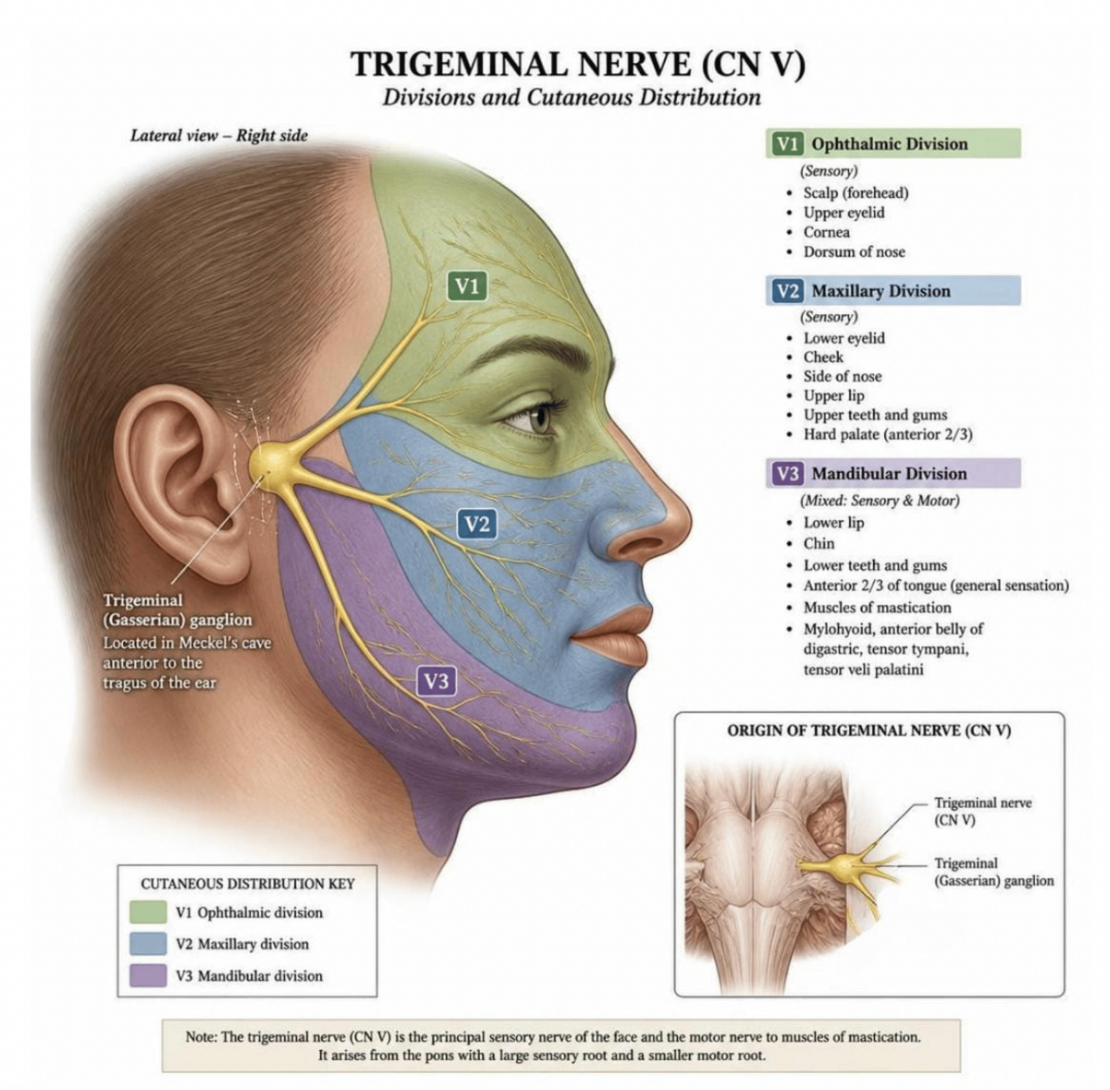
Schematic representation of the divisions of the trigeminal nerve (V) and their cutaneous distribution on the face - ophthalmic (V1), maxillary (V2), and mandibular (V3). In SUNCT (Short-lasting Unilateral Neuralgiform headache attacks with Conjunctival injection and Tearing), pain typically follows the V1 distribution, involving the periorbital and frontal regions, with associated autonomic symptoms (ipsilateral conjunctival injection and lacrimation). Illustration created by the authors using BioRender (BioRender Inc., Toronto, ON, Canada) and adapted from Kadunc et al. [10]. No generative artificial intelligence (AI) tools or AI-assisted features were used in the creation of this figure.

Short-lasting Unilateral Neuralgiform headache attacks with Conjunctival injection and Tearing (SUNCT) is among the most challenging trigeminal autonomic cephalalgias to manage [6], frequently displaying refractoriness to both pharmacological and surgical interventions [8]. Long-term longitudinal data are scarce, largely due to the low prevalence of the syndrome and the episodic nature of follow-up in most published reports. Symptom onset typically occurs at 40 years of age [8]; in our patient, onset occurred at 14 years. The diagnosis of SUNCT is based on well-established criteria, including a minimum number of attacks, specific pain characteristics, short duration, high frequency, and the presence of ipsilateral cranial autonomic symptoms [1]. Typically, patients experience strictly unilateral pain of moderate to severe intensity in trigeminal distributions, lasting from 1 to 600 seconds and occurring at least once daily. In addition, at least one autonomic feature, such as conjunctival injection, lacrimation, nasal congestion, or eyelid edema, is required, and alternative diagnoses, including trigeminal neuralgia, cluster headache, paroxysmal hemicrania, hemicrania continua, SUNA, and secondary trigeminal autonomic cephalalgias associated with structural lesions, must be excluded. The cutaneous distribution of the trigeminal nerve divisions is illustrated in Figure 3.

In the present case, the patient fulfilled all diagnostic criteria, presenting with frequent daily attacks of severe unilateral periorbital pain, lasting a few minutes and associated with prominent autonomic signs such as lacrimation, conjunctival injection, and eyelid edema. These findings strongly support the diagnosis of SUNCT and are consistent with previously described clinical patterns.

Neurovascular conflict has been increasingly recognized in SUNCT and other trigeminal autonomic cephalalgias [7]. Neurovascular contact between cerebellopontine arterial loops and the trigeminal nerve has been reported in a substantial proportion of SUNCT/SUNA patients, particularly when associated with morphological changes such as nerve displacement, distortion, or atrophy [11-14]. Although this situation may also be observed in asymptomatic individuals, these structural abnormalities appear to be more strongly associated with symptomatic disease and may help distinguish clinically relevant compression from incidental findings.

A large body of imaging and surgical evidence supports a role for neurovascular compression in at least a subset of patients [11,12,15]. Microvascular decompression (MVD) has been associated with complete or near-complete remission in approximately 70-75% of carefully selected cases, particularly when clear ipsilateral compression is demonstrated [14,16,17].

However, treatment failures and recurrences have also been reported. In the present patient, despite radiological evidence of neurovascular conflict and subsequent surgical intervention, clinical improvement was only transient, suggesting that neurovascular compression may act as a facilitating or perpetuating factor rather than the sole etiological mechanism of SUNCT [4,11,12,18].

The pathophysiology of SUNCT is increasingly understood as a complex interaction between peripheral trigeminal afferents and central modulatory structures. Current evidence supports the involvement of the trigeminal-autonomic reflex, in which activation of trigeminal nociceptive pathways triggers parasympathetic outflow via connections with the superior salivatory nucleus, resulting in the characteristic cranial autonomic features [5,19]. Neuroimaging studies across trigeminal autonomic cephalalgias have consistently demonstrated activation of the posterior hypothalamic region during attacks, suggesting a key role of hypothalamic dysregulation in the modulation of pain and autonomic responses [15].

In addition, SUNCT shares clinical and pathophysiological overlap with trigeminal neuralgia [20], particularly the presence of mechanical triggers and paroxysmal pain characteristics. This overlap has led to the hypothesis that SUNCT may represent a spectrum disorder within trigeminal pain syndromes, in which central dysfunction interacts with peripheral trigeminal hyperexcitability [5]. This integrated model may help explain the marked heterogeneity in clinical presentation and treatment response observed among SUNCT patients.

Cutaneous triggers within the trigeminal distribution, as observed in this patient, further reinforce this overlap with trigeminal neuralgia. Nevertheless, important clinical distinctions remain, suggesting that these entities may represent different manifestations along a shared pathophysiological spectrum.

This case also illustrates how treatment-related complications can significantly impact long-term outcomes. The development of painful anesthesia following trigeminal thermocoagulation represented a disabling condition nearly as burdensome as the original headache syndrome, underscoring the need for caution when considering destructive procedures, particularly in younger patients with a long disease trajectory. It is important to note that the exact radiofrequency modality and procedural approach could not be determined from the available historical medical records, and this limitation should be considered when interpreting the complication. 

Painful anesthesia (anesthesia dolorosa) is an uncommon but potentially devastating complication of destructive trigeminal procedures. It is believed to result from partial deafferentation of the trigeminal sensory pathways, leading to maladaptive central reorganization and spontaneous neuronal hyperactivity that produces persistent neuropathic pain despite objective sensory loss. This paradoxical combination of numbness and pain illustrates that interruption of peripheral afferent transmission does not necessarily abolish pain generation once central sensitization has become established. Consequently, destructive procedures should be reserved for carefully selected patients after a thorough discussion of the risk of irreversible neuropathic complications [21].

Beyond the trigeminal symptoms themselves, another notable finding was the occurrence of severe postoperative incisional pain following hysterosalpingo-oophorectomy, phenomenologically resembling trigeminal neuropathic pain and refractory to systemic opioids, yet responsive to local anesthetic blockade. This observation raises the hypothesis of central sensitization involving higher-order nociceptive pathways. Although not previously described in SUNCT, this phenomenon may reflect broader dysfunction in pain-modulation networks in chronic trigeminal autonomic cephalalgias.

Neuroimaging plays a critical role in evaluating SUNCT, particularly in identifying neurovascular conflict and excluding secondary causes. High-resolution magnetic resonance imaging with dedicated trigeminal nerve protocols is recommended, ideally combining 3D T2-weighted sequences, time-of-flight MR angiography, and contrast-enhanced T1-weighted imaging. These complementary techniques allow detailed visualization of neurovascular relationships and identification of morphological nerve changes, which are strongly associated with symptomatic cases and may guide surgical decision-making [12,20,22].

Pharmacological management in this case required a multimodal and individualized approach. Despite intolerance or refractoriness to lamotrigine, often considered first-line therapy, with reported response rates of up to 80% [2,23], sustained control was achieved through a combination of gabapentin, clonazepam, and targeted anesthetic nerve blocks. Although peripheral nerve blocks do not directly target the hypothalamic dysfunction believed to underlie SUNCT, they may interrupt nociceptive afferent input from trigeminal branches, thereby reducing activation of the trigeminocervical complex and the trigeminal-autonomic reflex. This transient modulation of peripheral input may attenuate central sensitization and decrease attack frequency and severity, explaining their usefulness as adjunctive therapy in selected refractory patients despite the predominantly central pathophysiology of the disorder [4,18]. The adjunctive use of onabotulinum toxin may have contributed to long-term stabilization, although its isolated effect cannot be determined. It is relevant to report that IV lidocaine and mexiletine are pharmacological options that were not tested in this patient due to the unavailability of these medications.

Long-term outcome data in SUNCT remain limited due to the rarity of the disorder and the predominance of case reports and small observational series [5,24]. Available cohort studies suggest that many patients require multiple therapeutic strategies over time, often experiencing fluctuating disease activity, incomplete remission, and changing treatment responses [5,24]. Even among patients who initially achieve satisfactory control with pharmacological therapy or microvascular decompression, recurrence may occur months or years later [18]. The prolonged disease course observed in the present patient, spanning several decades and involving multiple medical and surgical interventions, illustrates the chronic and relapsing nature of SUNCT and highlights the importance of long-term follow-up.

Microvascular decompression has demonstrated favorable outcomes in selected patients [12,14,16,25] with documented neurovascular conflict, particularly in those refractory to medical therapy. Reported success rates range from approximately 70% to nearly 90%, especially when clear radiological evidence of ipsilateral compression with morphological nerve changes is present. However, not all patients achieve sustained benefit, as illustrated in this case, reinforcing the notion that neurovascular compression alone does not fully explain the pathogenesis of the disease.

**Figure 4:**
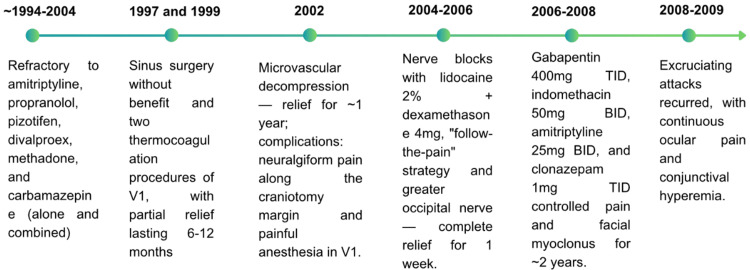
Chronological timeline of interventions, 1994-2009 Source: Created by the authors.

**Figure 5:**
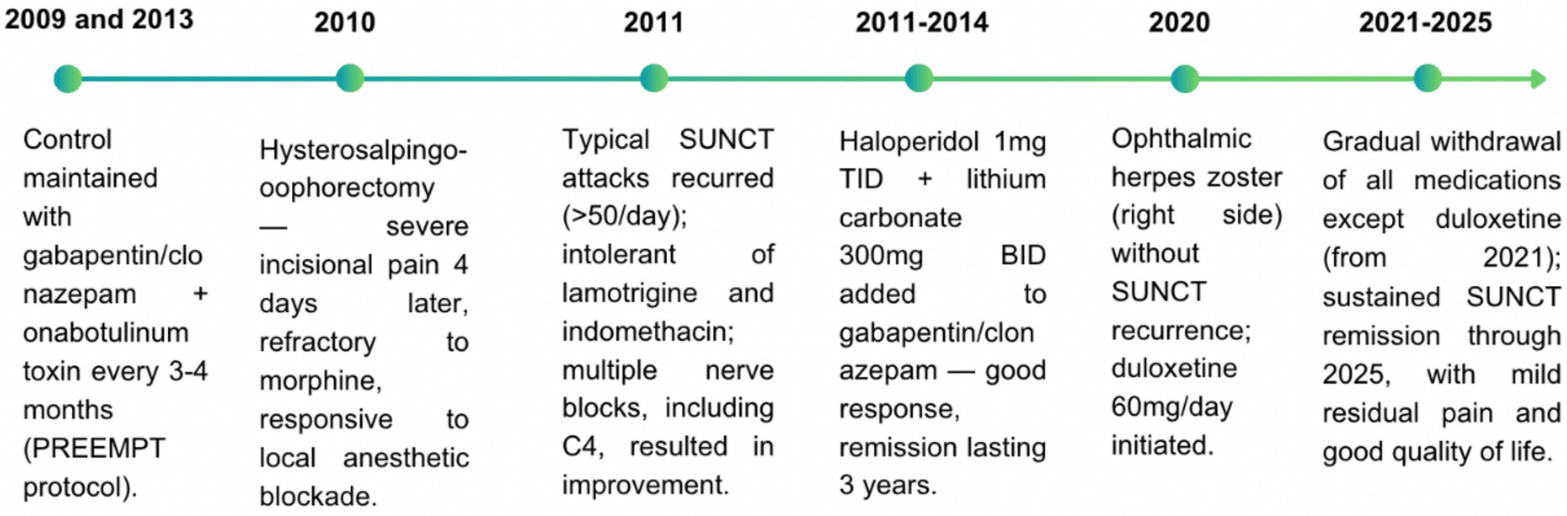
Chronological timeline of interventions, 2009-2025 Source: Created by the authors. PREEMPT protocol - Blumenfeld et al. [9]. SUNCT: Short-lasting Unilateral Neuralgiform headache attacks with Conjunctival injection and Tearing

Overall, this case highlights the chronic and complex nature of SUNCT, the limitations of purely peripheral or surgical approaches, and the importance of long-term multimodal management strategies [24]. Interprofessional collaboration is essential to mitigate the disease burden, and extended follow-up is crucial to better understand disease evolution, treatment response, and late-emerging complications. The chronology of the patient's clinical course and therapeutic interventions is summarized in Figure 4 and Figure 5. 

## Conclusions

This case represents one of the longest reported follow-ups of a patient with Short-lasting Unilateral Neuralgiform headache attacks with Conjunctival injection and Tearing (SUNCT), documenting a 21-year disease course of a rare disorder characterized by multiple periods of remission and recurrence. The longitudinal observation revealed significant resistance to various pharmacological treatments, including lamotrigine, and serious complications related to these therapies. Long-term disease control was achieved through a personalized, multimodal management strategy and consistent follow-up. This report offers insight into the long-term evolution of SUNCT and emphasizes the importance of careful therapeutic decision-making and ongoing observation in patients with a rare primary headache.

## References

[1] (2018). Headache classification committee of the International Headache Society (IHS). The international classification of headache disorders, third edition. Cephalalgia.

[2] Cohen AS, Matharu MS, Goadsby PJ (2006). Short-lasting unilateral neuralgiform headache attacks with conjunctival injection and tearing (SUNCT) or cranial autonomic features (SUNA): a prospective clinical study of SUNCT and SUNA. Brain.

[3] Hagen K, Tronvik E, Stovner LJ (2024). One-year prevalence of cluster headache, hemicrania continua, paroxysmal hemicrania, and SUNCT in Norway: a population-based nationwide registry study. J Headache Pain.

[4] Goadsby PJ, Holland PR, Martins-Oliveira M (2017). Pathophysiology of trigeminal autonomic cephalalgias. Physiol Rev.

[5] Duggal AK, Chowdhury D (2021). SUNCT and SUNA: an update. Neurol India.

[6] Chowdhury D (2018). Secondary (symptomatic) trigeminal autonomic cephalalgias. Ann Indian Acad Neurol.

[7] Coo IF, Wilbrink LA, Haan J (2015). Symptomatic trigeminal autonomic cephalalgias. Curr Pain Headache Rep.

[8] Ali M, Khan S, Hussain N (2022). Refractory short-lasting unilateral neuralgiform headache attacks with conjunctival injection and tearing (SUNCT) responding to erenumab adjuvant therapy: a case report. Cureus.

[9] Blumenfeld A, Silberstein SD, Dodick DW, Aurora SK, Turkel CC, Binder WJ (2010). Method of injection of onabotulinumtoxinA for chronic migraine: a safe, well-tolerated, and effective treatment paradigm based on the PREEMPT clinical program. Headache.

[10] Kadunc BV, Palermo EC, Addor FA, Metsavaht L, Rabello L, Mattos R (2012). Tratado de cirurgia dermatológica, cosmiatria e laser da Sociedade Brasileira de Dermatologia. laser da Sociedade Brasileira de Dermatologia. Rio de Janeiro: Elsevier.

[11] Lambru G, Rantell K, O’Connor E (2020). Trigeminal neurovascular contact in SUNCT and SUNA: a cross-sectional magnetic resonance study. Brain.

[12] Favoni V, Grimaldi D, Pierangeli G, Cortelli P, Cevoli S (2013). SUNCT/SUNA and neurovascular compression: new cases and critical literature review. Cephalalgia.

[13] Kitahara I, Fukuda A, Imamura Y, Ikawa M, Yokochi T (2015). Pathogenesis, surgical treatment, and cure for SUNCT syndrome. World Neurosurg.

[14] Hassan S, Lagrata S, Levy A, Matharu M, Zrinzo L (2018). Microvascular decompression or neuromodulation in patients with SUNCT and trigeminal neurovascular conflict?. Cephalalgia.

[15] Sprenger T, Valet M, Platzer S (2005). SUNCT: bilateral hypothalamic activation during headache attacks and resolving of symptoms after trigeminal decompression. Pain.

[16] Barazi S, Pak HL, Maratos E, Connor S, Lambru G (2025). Trigeminal microvascular decompression for medically refractory short-lasting unilateral neuralgiform headache attacks: a single-center retrospective analysis. World Neurosurg.

[17] Williams M, Bazina R, Tan L, Rice H, Broadley SA (2010). Microvascular decompression of the trigeminal nerve in the treatment of SUNCT and SUNA. J Neurol Neurosurg Psychiatry.

[18] Lambru G, Matharu MS (2014). SUNCT, SUNA and trigeminal neuralgia: different disorders or variants of the same disorder?. Curr Opin Neurol.

[19] Goadsby PJ (2005). Trigeminal autonomic cephalalgias. Pathophysiology and classification. Rev Neurol (Paris.

[20] Lambru G, Trimboli M, Tan SV, Al-Kaisy A (2017). Medullary infarction causing coexistent SUNCT and trigeminal neuralgia. Cephalalgia.

[21] Nurmikko TJ, Eldridge PR (2001). Trigeminal neuralgia--pathophysiology, diagnosis and current treatment. Br J Anaesth.

[22] Coskun O, Ucar M, Vuralli D (2017). MR tractography in short-lasting unilateral neuralgiform headache attacks with conjunctival injection and tearing (SUNCT) patients: case reports. Pain Med.

[23] Bendtsen L, Zakrzewska JM, Abbott J (2019). European Academy of Neurology guideline on trigeminal neuralgia. Eur J Neurol.

[24] Cohen AS (2007). Short-lasting unilateral neuralgiform headache attacks with conjunctival injection and tearing. Cephalalgia.

[25] Al Khalili Y, Veerapaneni KD (StatPearls [Internet]. StatPearls Publishing). Short-lasting unilateral neuralgiform headache. In. https://www.ncbi.nlm.nih.gov/books/NBK559088/.

